# The Nature and Origin of Synaptic Inputs to Vestibulospinal Neurons in the Larval Zebrafish

**DOI:** 10.1523/ENEURO.0090-23.2023

**Published:** 2023-06-01

**Authors:** Kyla R. Hamling, Katherine Harmon, David Schoppik

**Affiliations:** Departments of Otolaryngology and Neuroscience & Physiology, and Neuroscience Institute, New York University Grossman School of Medicine, New York, New York 10016

**Keywords:** balance, electrophysiology, evolution, vestibulospinal

## Abstract

Vestibulospinal neurons integrate sensed imbalance to regulate postural reflexes. As an evolutionarily conserved neural population, understanding their synaptic and circuit-level properties can offer insight into vertebrate antigravity reflexes. Motivated by recent work, we set out to verify and extend the characterization of vestibulospinal neurons in the larval zebrafish. Using current-clamp recordings together with stimulation, we observed that larval zebrafish vestibulospinal neurons are silent at rest, yet capable of sustained spiking following depolarization. Neurons responded systematically to a vestibular stimulus (translation in the dark); responses were abolished after chronic or acute loss of the utricular otolith. Voltage-clamp recordings at rest revealed strong excitatory inputs with a characteristic multimodal distribution of amplitudes, as well as strong inhibitory inputs. Excitatory inputs within a particular mode (amplitude range) routinely violated refractory period criteria and exhibited complex sensory tuning, suggesting a nonunitary origin. Next, using a unilateral loss-of-function approach, we characterized the source of vestibular inputs to vestibulospinal neurons from each ear. We observed systematic loss of high-amplitude excitatory inputs after utricular lesions ipsilateral, but not contralateral, to the recorded vestibulospinal neuron. In contrast, while some neurons had decreased inhibitory inputs after either ipsilateral or contralateral lesions, there were no systematic changes across the population of recorded neurons. We conclude that imbalance sensed by the utricular otolith shapes the responses of larval zebrafish vestibulospinal neurons through both excitatory and inhibitory inputs. Our findings expand our understanding of how a vertebrate model, the larval zebrafish, might use vestibulospinal input to stabilize posture. More broadly, when compared with recordings in other vertebrates, our data speak to conserved origins of vestibulospinal synaptic input.

## Significance Statement

Vestibulospinal neurons are an ancient vertebrate cell type that integrates sensed instability to guide balance. Recent work (Liu et al., 2020) characterized the synaptic inputs and spiking outputs of larval zebrafish vestibulospinal neurons, and proposed a model for this transformation. First, we validate these findings, reinforcing the utility of the larval zebrafish model for understanding postural control. We then establish the laterality of excitatory and inhibitory synaptic inputs, allowing comparison of vestibulospinal circuit architecture in larval zebrafish to other vertebrate species. Together, our work establishes functional consensus and evolutionary context for zebrafish vestibulospinal neurons, a key step toward understanding vertebrate postural stabilization.

## Introduction

Vestibular reflexes maintain posture in the face of gravity (Goldberg et al., 2012). These reflexes originate with evolutionarily ancient “vestibulospinal” circuits that link the vestibular sensory periphery and the spinal cord (Boyle, 2020; Zhu et al., 2020). Vestibulospinal neurons, first identified by Deiters (Voogd, 2016), are descending projection neurons found in the lateral vestibular nucleus of the hindbrain. Defining the properties of synaptic inputs to vestibulospinal neurons is critical to understand how sensed imbalance is transformed into corrective behaviors.

The larval zebrafish has emerged as a useful model for studying balance behaviors (Bagnall and Schoppik, 2018), particularly those mediated by vestibular circuits (Bianco et al., 2012; Ehrlich and Schoppik, 2017; Favre-Bulle et al., 2017, 2018; Schoppik et al., 2017; Migault et al., 2018; Tanimoto et al., 2022; Beiza-Canelo et al., 2023; Hamling et al., 2023; Sugioka et al., 2023). At 4 d postfertilization (dpf), larval zebrafish maintain a dorsal-up stable roll posture to navigate in the water column, find food, and avoid predators. To do so, loss-of-function experiments (Riley and Moorman, 2000; Mo et al., 2010; Roberts et al., 2017) suggest they rely on a sense of gravity mediated by an otolithic (utricular) organ; while present and capable of transduction (Goldblatt et al., 2023), their semicircular canals are too small to function under normal conditions (Lambert et al., 2008). Larval zebrafish are genetically tractable and largely transparent, allowing for rapid and reliable identification of vestibulospinal neurons (Kimmel et al., 1982; Liu et al., 2020). Unlike most other preparations, larval zebrafish vestibulospinal neurons are accessible for *in vivo* patch-clamp recording allowing characterization of their synaptic inputs.

Recent work (Jia and Bagnall, 2022; Liu et al., 2022, 2020) suggests a model for how synaptic inputs onto vestibulospinal neurons might shape their response. Vestibular neuron responses are largely linear—a feature thought to facilitate proportional and continuous reflexive responses to destabilization (but see Jamali et al., 2019). Unlike most sensory synapses that either adapt or facilitate, the synapse between peripheral afferents and brainstem vestibular neurons has a number of specializations, identified with slice electrophysiology and electron micrography, to allow linear transmission (Bagnall et al., 2008; McElvain et al., 2015). These specializations predict that *in vivo* release from individual vestibular afferents might produce depolarization with a characteristic amplitude in a target vestibulospinal neuron. Recent findings by Liu et al. (2020) support this model: excitatory synaptic inputs to vestibulospinal neurons had remarkably stereotyped amplitudes. Further, Liu et al. (2020) performed genetic loss-of-function experiments that suggest a dominant role for utricular inputs in driving vestibulospinal responses to translation. Follow-up experiments complement these loss-of-function findings with hemibrain EM datasets that establish synaptic connectivity between ipsilateral otolithic afferents and vestibulospinal neurons (Jia and Bagnall, 2022; Liu et al., 2022). To date, the electrophysiological and loss-of-function findings have neither been replicated nor extended.

Vertebrates use vestibular sensory organs in each of two ears to detect imbalance. Vestibulospinal neurons could therefore receive unilateral and/or bilateral input, and this input could be excitatory or inhibitory. Comparing information across ears is key to proper vestibular behavior (Markham et al., 1977; Malinvaud et al., 2010; Dieterich and Brandt, 2015), as revealed following unilateral loss of VIIIth nerve input (Black et al., 1989; Halmagyi et al., 2010). In particular, contralateral inhibition of broad origin (Shimazu and Precht, 1966), or restricted to the utricle (Uchino et al., 1999, 1986), has been proposed as a way to increase the sensitivity of central vestibular neurons. Intriguingly, there is an existing anatomic divide among vertebrates (e.g., between *Hyperoartia* and *Mammalia*) regarding the lateralization of excitatory and inhibitory input (Zhu et al., 2020). To date, only ipsilateral excitatory input has been characterized in larval zebrafish vestibulospinal neurons (Liu et al., 2020), leaving open questions of vestibulospinal circuit homology and function.

In this article, we investigated the nature and origin of synaptic input onto vestibulospinal neurons in larval zebrafish. We began by validating and extending three key findings. First, we observed that vestibulospinal neurons, while silent at rest, can fire sustained trains of action potentials. We used both acute and chronic loss-of-function approaches to establish that phasic responses of vestibulospinal neurons to translation in the dark originate with the utricle. We then used voltage-clamp recordings to characterize the spontaneous excitatory (as done before) and inhibitory (novel) synaptic inputs. While excitatory synaptic inputs on vestibulospinal neurons were separable into discrete event amplitudes, in most cases these failed a refractory period test. We then used unilateral lesions to map the organization of spontaneous synaptic inputs to vestibulospinal neurons. We found that, like mammalian central vestibular circuits, high-amplitude excitatory inputs derive from the ipsilateral ear, whereas inhibitory inputs originate from both the ipsilateral and contralateral ear. Together, our work builds on and extends previous findings to characterize the synaptic inputs to vestibulospinal neurons in the larval zebrafish. The similarities we observe to mammalian architecture and the ability to replicate basic findings across laboratories solidify the utility of the larval zebrafish to understand the synaptic computations that mediate vestibulospinal reflexes so crucial for vertebrate balance.

## Materials and Methods

### Fish care

All procedures involving zebrafish larvae (*Danio rerio*) were approved by the Institutional Animal Care and Use Committee of New York University. Fertilized eggs were collected and maintained at 28.5°C on a standard 14 h light/10 h dark cycle. Before 5 dpf, larvae were maintained at densities of 20–50 larvae/10-cm-diameter Petri dish, filled with 25–40 ml of E3 with 0.5 ppm methylene blue. After 5 dpf, larvae were maintained at densities under 20 larvae/Petri dish and were fed cultured rotifers (Reed Mariculture) daily.

### Fish lines

Experiments were done on the *mitfa*^−/−^ background to remove pigment. For chronic bilateral utricular lesions, fish with a homozygous recessive loss-of-function mutation of the inner ear-restricted gene, *otogelin* (otog^−/−^), previously called *rock solo*^AN66^ (Whitfield et al., 1996), were visually identified by a lack of utricular otoliths.

### Electrophysiology

Larval zebrafish used for experiments were between 3 and 12 dpf in age, with most fish being between 4 and 7 dpf (3 dpf, *N* = 1; 4 dpf, *N* = 19; 5 dpf, *N* = 21; 6 dpf, *N* = 9; 7 dpf, *N* = 5; 8 dpf, *N* = 2; 9 dpf, *N* = 1; 12 dpf, *N* = 1). Ages of fish used in each condition are listed in Table 1. Fish were paralyzed with pancuronium bromide (0.6 mg/ml) in external solution (in mm: 134 NaCl, 2.9 KCl, 1.2 MgCl_2_, 10 HEPES, 10 glucose, and 2.1 CaCl_2_) until movement ceased. Fish were then mounted dorsal-up in 2% low-melting temperature agarose, and a small incision was made in the skin above the cerebellum. Pipettes (impedance, 7–9 MΩ) were lowered to the plane of the Mauthner cell body, illuminated by infrared (900 nm) differential interference contrast optics. Putative vestibulospinal neurons were targeted for patching using soma size and proximity to the Mauthner lateral dendrite as guides. Initial targeting conditions were determined with reference to cells labeled by spinal backfills. Subsequent recordings were determined to be from vestibulospinal neurons by postrecording analysis of anatomic morphology, confirming a single ipsilateral descending axon using either wide-field fluorescence or confocal microscopy.

**Table 1:** Spontaneous properties across conditions

	Unit	Control	Cs control	Ipsi lesion	Contra lesion	Bilateral lesion	*otogelin*
Resting membrane potential	mV	−67.3	−60.8	−63.7	−63.8	−68.9	−66
Series resistance	MΩ	34.7	25.8	43.6	45.7	38.4	31.2
Input resistance	MΩ	236	202	264	178	310	228
Resting firing frequency	Hz	1.4				0	0
Rheobase	pA	111.8				38.9	58.8
Resting EPSC frequency	Hz	101.3	65.4	30.2	59.2	46.4	119.2
Age range	dpf	3–12	4–5	4–5	4–6	5	5–6

Ipsi, Ipsilateral; Contra, contralateral; Cs, Cesium.

All electrophysiological measurements were made in the dark. Pipettes were filled with dye (Alexa Fluor 647 hydrazide; catalog #A20502, Thermo Fisher Scientific) in the internal solution (in mm: 125 K-gluconate, 3 MgCl_2_, 10 HEPES, 1 EGTA, and 4 Na_2_-ATP). For recordings with voltage-clamp trials at 0 mV holding potential, pipettes were filled with a Cesium-based internal solution to prevent action potentials (in mm: 122 CsMeSO_3_, 5 QX-314 Cl, 1 TEA-Cl, 3 MgCl_2_, 10 HEPES, 1 EGTA, and 4 Na_2_-ATP). During trials to determine rheobase and maximum firing frequency, cells were injected with three pulses (pulse duration, 0.5 s) of decreasingly hyperpolarizing current, and seven pulses (pulse duration, 0.5 s) of increasingly depolarizing current. The magnitude of current steps was scaled for each cell until spikes were seen during the strongest depolarizing current. The average current step size for control vestibulospinal neurons was 21.75 pA (range, 12–46 pA) with an average peak depolarizing current of 152.25 pA (range, 84–322 pA). In current-clamp trials during linear acceleration stimuli, cells were often injected with an offset depolarizing current (range, 0–292.7 pA; mean, across conditions, 46.8 pA; control, 57.6 pA; acute bilateral utricle removal, 32.8 pA; chronic bilateral utricle removal, 23.8 pA) until spontaneous action potentials were seen during the baseline period without translation.

### Linear translation stimulus

Fish were mounted dorsally on an air table (Technical Manufacturing Corporation) with handles (catalog #55025A51, McMaster-Carr) mounted underneath for manual translation. The air table was then pushed/pulled manually in either the lateral or fore/aft direction to produce table oscillations that were smoothed because of the inherent resistance of the air table. Acceleration traces of table oscillations were measured using a three-axis accelerometer (catalog #ADXL335, SparkFun) mounted to the air table. Table oscillations persisted for an average of 11.69 ± 5.05 s, with a mean frequency of 1.54 ± 0.17 Hz and a peak acceleration of 0.91 ± 0.21 × *g* across all trials (*n* = 122 trials; (lateral, 73 trials; fore–aft, 49 trials). Lateral translation trials were longer in duration than fore–aft trials (13.02 s lateral vs 9.71 s fore–aft; *p* = 2.98 × 10^−4^ unpaired *t* test), and higher in peak acceleration (1.01 × *g* lateral vs 0.75 × *g* fore–aft; *p* = 3.02 × 10^−14^), but did not significantly differ in stimulus frequency from fore–aft stimuli (1.52 Hz lateral vs 1.58 Hz fore–aft; *p* = 0.051).

### Electrophysiology analysis

Data analysis and modeling were performed using MATLAB (MathWorks). For both voltage-clamp and current-clamp recordings, events were first selected using an automatic initial threshold that was set to 15% of the current or voltage range for a given trial. On a minority of trials, this automatic threshold was changed by the experimenter to more accurately select events. All event selections were then confirmed by eye and additional events were hand selected or deselected as necessary. Postsynaptic current events were identified by eye as events with a waveform consisting of an initial sharp amplitude rise and exponential-like decay. Event assignations were made by a first experimenter and then checked by a second experimenter to ensure accurate event selection and to decrease experimenter selection bias. Manually verifying event selections ensured the highest confidence in detection accuracy. Exact event times were further identified by local minima or maxima search around hand-selected event times.

Action potential amplitude was calculated as the difference between peak membrane voltage and a baseline voltage (1 ms before event peak). The rheobase for vestibulospinal action potential generation was calculated by fitting a line to firing responses as a function of injected current, limited to all current steps above the minimum current injection that elicited spikes. The linear fit was used to solve for the estimated current necessary for each vestibulospinal neuron to fire at 1 Hz.

EPSC amplitudes were determined by subtracting the minima of the event waveform from a pre-event baseline current (0.4 ms before event minima). For EPSCs, amplitude “bins” were assigned manually using the probability distributions of EPSC amplitudes across all trials from the same cell. EPSCs with amplitudes <5 pA were excluded from analyses. IPSC amplitudes were determined by taking the difference between the maxima of the event waveform and a pre-event baseline current (2 ms before event maxima).

#### Assessment if EPSCs had a unitary origin

To determine whether EPSC bins were derived from a single afferent origin, we calculated the number of within-bin EPSCs that occurred within a 1 ms refractory period. We excluded events that occurred within 0.3 ms of each other from this analysis as manual inspection found that these were usually double selections of the same synaptic event, rather than two separable events. We only rarely observed vestibulospinal EPSC amplitude bins with zero within-bin violations. To minimize type II errors from overly strict refractory period violation criteria, we modeled an upper limit on the number of within-bin refractory period violations that we would expect to see from the overlap in EPSC amplitude distributions: the probability distribution of EPSC amplitudes (*I*) of each cell was estimated as a sum of Gaussian distributions, where the number of distributions was set to the number of EPSC amplitude bins in that cell. Each amplitude bin was fit with three free parameters for height (*h*), center (*c*) in pA, and SD (*σ*), as follows:

(1) $$p(I_{\text{A}})=he^{\frac{-{\left(I-c\right)}^{2}}{2\sigma^{2}}}.$$

Bin centers (*c*) were constrained to fall within bin amplitude cutoffs. For cells with only one amplitude bin or for the highest amplitude bin, *c* was constrained at the upper bound to be 1 SD above the peak amplitude probability. Bin heights (*h*) were constrained to be at least half of the maximum value of the empirical probability distribution within the amplitude bin limits. For a given bin of interest (*A*), we used the modeled probability distributions to calculate the number of expected false-positive refractory period violations [an across-bin event pair being falsely counted as a within-bin event pair (*ϕ*_A_)] according to the following formula:

(2) $$\phi_{\text{A}}=\beta_{\text{A}}(1.4\cdot H_{\text{A}}\cdot F_{\text{A}}),$$

where *β*_A_ is the number of observed EPSC events falling with amplitude bin *A* (defined by EPSC amplitude thresholds from *a* to *b* pA), $H_{\text{A}}$ is the hit event rate (events per millisecond assigned to bin *A* that truly derived from bin *A*), and $F_{\text{A}}$ is the false-positive event rate for bin *A* (events per millisecond falsely assigned to bin *A* when they derived from the overlapping tails of Gaussian distributions from other bins). The joint probability of $H_{\text{A}}$ and $F_{\text{A}}$ was multiplied by 1.4 to account for the 0.7 ms window preceding or following any given event to be counted as a refractory period violation (from 0.3 to 1 ms following, or −1 to −0.3 ms preceding). $H_{\text{A}}$ was calculated using the following:

(3) $$H_{\text{A}}=\frac{\beta \cdot \int_{a}^{b}p(I_{\text{A}})dI}{s},$$

where *β* is the total number of observed EPSC events across all amplitude bins, and *s* is the trial length in milliseconds. $F_{\text{A}}$ was calculated using the following:

(4) $$F_{\text{A}}=\frac{\beta \cdot \int_{a}^{b}p(I_{\text{B}})dI+\int_{a}^{b}p(I_{\text{C}})dI+...+\int_{a}^{b}p(I_{\text{N}})dI}{s},$$

where $p(I_{\text{B}})$ is the estimated probability distribution of the second EPSC amplitude bin, and $p(I_{\text{N}})$ is the *n*th EPSC amplitude bin.

For each amplitude bin in the cell, *ϕ* was calculated and compared with the number of observed within-bin violations. To determine whether bins with few to no observed refractory period violations occurred solely because of low event frequency, we calculated the number of expected refractory period violations in frequency-matched randomly generated event trains. EPSC bins were classified as unitary afferent bins if the number of empirical within-bin refractory period violations was fewer than the number of expected violations from bin overlap (*ϕ*) and if the frequency-matched generated controls had at least one observed violation.

#### Quantification of sensory responses

Instantaneous spike or EPSC rates were estimated by computing a peristimulus time histogram (PSTH) with a time bin width of one-sixteenth of the oscillation cycle length. The spiking response of a cell (or the EPSC response of an amplitude bin) was defined as the average PSTH across all stimulus cycles in a particular direction (lateral or fore–aft). Modulation depth was determined from this response, defined as the difference between the peak spiking rate and the minimum spiking rate. Cells were considered “directional” if their sensitivity was greater than 2 SDs from the mean modulation depth derived from 100 randomly generated frequency-matched spike trains. The number of firing rate or EPSC rate peaks per oscillation was determined by finding local maxima in the average PSTH. Local maxima were considered a true peak for the firing rate if they were >2 SDs from the shuffled mean modulation depth and phase shifted at least 90° from another true peak. Local maxima were considered a true peak for the EPSC rate if they were >1 SD above the mean prestimulus baseline EPSC rate for that amplitude bin and phase shifted at least 90° from another true peak. A cell (for firing rate) or amplitude bin (for EPSC rate) was considered to have “simple” tuning if it had only a single peak in lateral or fore–aft translation, and “complex” if it had more than one peak in a translation direction. For spiking rate, simple/complex tuning was determined separately for lateral and fore–aft directions in cells. For EPSC rate, simple/complex tuning for a single bin was determined by responses to both lateral and fore–aft directions; an amplitude bin that was complexly tuned in either direction was considered complexly tuned overall. EPSC bins that had no significant tuning (0 EPSC peaks) in either direction were excluded from this analysis (*n* = 1 amplitude bin).

### Utricular lesions

Chronic bilateral utricular lesions were achieved using mutant larvae (*otogelin*) that do not express *otogelin* and do not develop utricles until 11–12 dpf. Acute ipsilateral and contralateral lesions were performed using forceps to rupture the otic capsule and remove the utricular otolith from one ear. Acute lesions were performed on fish that were between 4 and 6 dpf, when the utricular otolith is ∼50 μm in diameter (Favre-Bulle et al., 2017) and can be removed with fine forceps (catalog #5CO, Dumont). This physically removes the sensory organ itself and likely damages the closely apposed hair cells in the utricular maculae whose spontaneous activity influences vestibular afferents. Acute bilateral lesions were performed through microinjection of 1 mm CuSO_4_ into both otic capsules to kill hair cells (Hernández et al., 2006), with coinjection of 40 μm FM 1–43 dye (catalog #T3163, Thermo Fisher Scientific) to label hair cell membranes for visualization. After ipsilateral utricle removal and bilateral copper injection, we saw a marked decrease in spontaneous inputs onto vestibulospinal neurons (Table 1), supporting the hypothesis that these lesions work to impair the firing rate of utricular vestibular afferents. Acute lesions may also impair inner ear function by diluting the potassium-rich ionic composition of ear endolymph that is critical for hair cell function (Köppl et al., 2018).

### Statistics

The expected value and variance of data are reported as the mean and the SD or the median and the median absolute difference. When data satisfied the criteria of normality (Lilliefors test for normality), parametric statistical tests were used, otherwise we used their nonparametric counterparts. Criteria for significance was set at 0.05 and, when applicable, corrected for multiple comparisons.

### Data availability

All raw data and code for analysis are available at the Open Science Framework (doi:10.17 605/OSF.IO/M8AG9).

## Results

### Vestibulospinal neurons encode body translation using utricular sensory inputs

**Figure 1.**
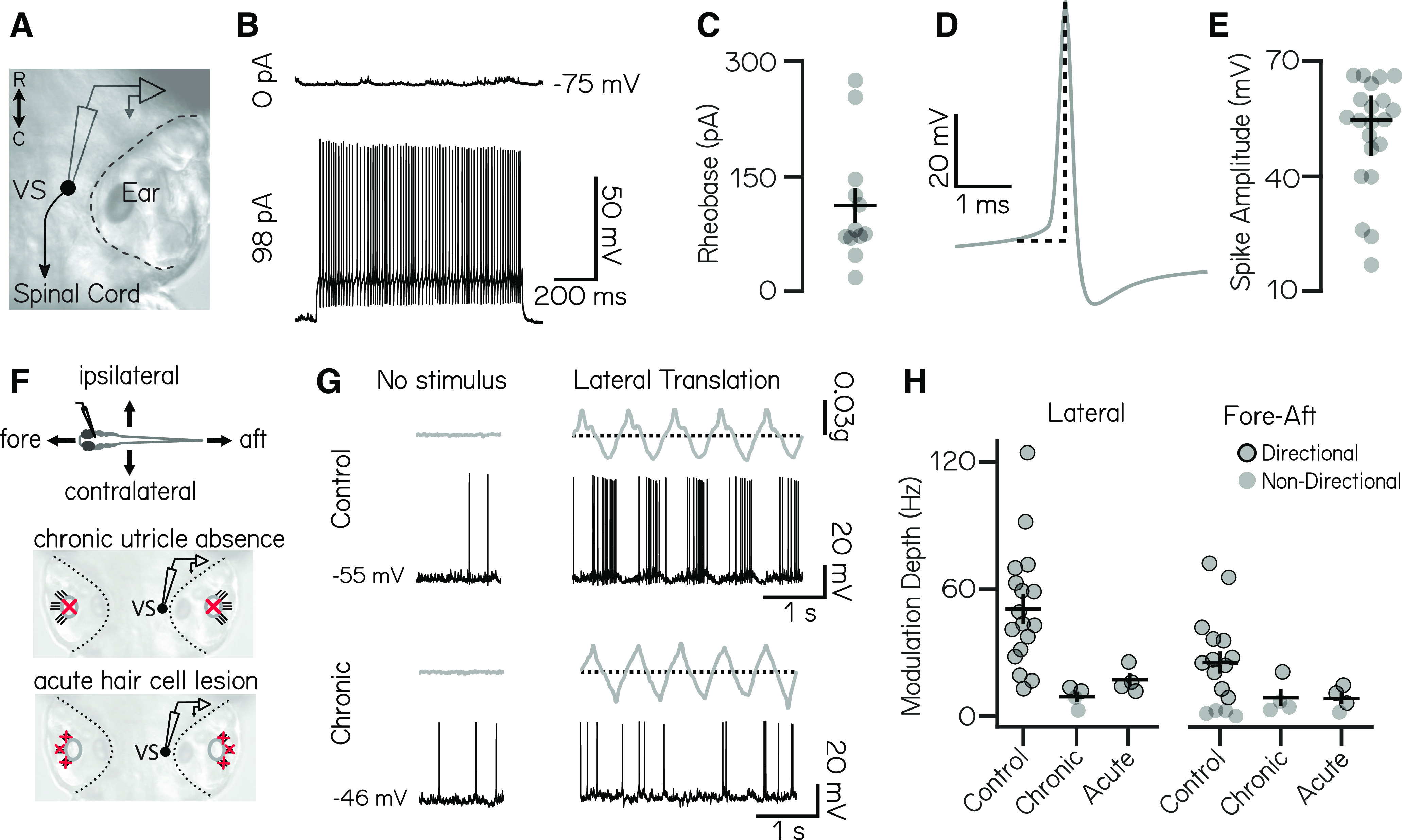
Vestibulospinal neurons encode utricle-derived body translation. ***A***, Schematic of spinal-projecting vestibulospinal (VS) neuron targeted for electrophysiology. ***B***, Vestibulospinal membrane potential at rest (top, 0 pA) and in response to current injection (bottom, 98 pA). ***C***, Rheobase (mean ± SEM) across 12 nonspontaneously active cells. ***D***, Example action potential waveform with amplitude (dotted line). ***E***, Action potential average amplitude (median ± interquartile range) across 21 cells. ***F***, Immobilized fish were manually translated in the fore–aft or lateral axes (top). Vestibulospinal neurons were recorded in control and after two manipulations: first, in *otogelin* mutants (middle) that do not develop utricles (red “x”) and second, after chemically induced hair cell (red “x”) death (bottom). ***G***, Accelerometer (gray) and voltage trace (black) from a neuron in a control fish (top) showing action potentials in phase with translation. In contrast, activity from an *otogelin* mutant (bottom) is unaligned with translation. ***H***, Modulation depth of spiking response (mean ± SEM) is disrupted in both the lateral (left) and fore–aft (right) directions after both chronic and acute disruption of the utricle. Gray circles, neurons; black outlined circles, statistically significant directional responses.

We first characterized the basic electrophysiolgical properties of larval zebrafish vestibulospinal neurons. We performed *in vivo* whole-cell patch-clamp recordings in the dark in fish that were 3–12 dpf (*n* = 21 cells; Fig. 1*A*). For all experiments, we did not see a significant effect of age on recording properties; therefore, results have been combined across ages. Only one neuron was recorded per fish; henceforth, we will report only the number of cells recorded per experiment. Dye in the recording solution allowed *post hoc* confirmation of vestibulospinal identity by visualization of descending axons. Neurons had high input resistance (236 ± 130 MΩ) and resting membrane potential of −67 ± 5 mV (Table 1). At rest, approximately half of vestibulospinal neurons (*n* = 12 of 21) showed no spontaneous action potentials (Fig. 1*B*, top); the remaining 9 had a median firing rate at rest of 0.9 Hz (range, 0.02–16.3 Hz). Silent vestibulospinal neurons could, however, sustain a high tonic firing rate (90.3 ± 68.5 Hz) following current injection (largest depolarizing step per cell, 84–322 pA; Fig. 1*B*, bottom). Qualitatively, all neurons displayed tonic firing when driven by current steps substantially above their rheobase. The high rheobase of 111.8 ± 78.9 pA (Fig. 1*C*) in cells that were silent at rest suggests the lack of spontaneous firing activity reflects a high spiking threshold in these neurons. Action potentials had a stereotyped waveform with a median spike amplitude of 54.6 mV (Fig. 1*D*,*E*) and a single afterhyperpolarization phase (mean, 5.7 ± 2.6 mV). We conclude that, similar to *Xenopus* vestibular neurons (Straka and Dieringer, 1996; Straka et al., 2004), larval zebrafish vestibulospinal neurons are largely silent at rest but capable of firing sustained trains of action potentials.

We next assayed the responses of vestibulospinal neurons to sensory stimulation. We provided an oscillatory translation—in the dark—either along the fore–aft or lateral axis of the fish during whole-cell recordings (Fig. 1*F*, top). To ensure that we did not miss evoked responses because of insufficient sensory stimulation, when recording from neurons that were completely silent at rest (*n* = 9 of 17 cells), we injected a small bias current (91.9 ± 79.6 pA), following previously published methods (Liu et al., 2020). The presence or absence of this depolarizing current did not affect further conclusions, and so all cells were combined for analysis.

We observed that vestibulospinal neurons fired phasically during translation (Fig. 1*G*, top, response to example lateral translation). In both lateral and fore–aft directions, the majority of cells had a single peak in firing rate during an oscillation cycle and a single antiphase firing pause (simple tuning: lateral, 15 of 17; fore–aft, 10 of 16), but a small percentage of cells had multiple peak/pauses in firing (complex tuning: lateral, 2 of 17; fore–aft, 2 of 16) as had previously been observed in the fore–aft axis (Liu et al., 2020). We quantified the directional tuning of a cell in each translational axis (lateral or fore–aft) by calculating the difference between the peak firing rate and the minimum firing rate during an oscillation cycle (referred to as the “modulation depth”; Materials and Methods; Dietrich et al., 2017). This method performed best qualitatively for analyzing both simple and complex tuning cells and was strongly correlated with comparable tuning metrics such as taking the difference between peak phasic response and the 180° antiphase response (Pearson’s correlation, *ρ* = 0.99). We then tested for statistically significant directional tuning by comparing the modulation depth of a cell for that stimulus relative to that derived from randomly shuffled data. We subsequently categorized each cell as “directional” or “nondirectional” for each axis. All recorded neurons (17 of 17) responded directionally to lateral stimuli (modulation depth, 50.6 ± 28.6 Hz), and most neurons (12 of 16) were directionally responsive to fore–aft stimuli (modulation depth, 25.3 ± 21.7 Hz; Fig. 1*H*, directional neurons circled). Most neurons were directionally tuned to the peak acceleration of the stimulus toward the contralateral side (6 of 10 neurons) and rostral direction (7 of 10 neurons). We conclude that the activity of vestibulospinal neurons can encode translation.

Previous loss-of-function studies established that the utricle is the dominant source of sensory information about body tilts in larval zebrafish (Riley and Moorman, 2000; Mo et al., 2010; Bianco et al., 2012). We asked whether the evoked responses we observed reflected activity originating in the utricular macula. We adopted a loss-of-function approach, recording from vestibulospinal neurons in *otogelin* mutants that fail to develop utricles (Fig. 1*F*, chronic utricle absence; Whitfield et al., 1996). Neurons in mutant fish could still fire action potentials but failed to respond phasically during body translation (Fig. 1*G*, bottom). Modulation depth was decreased in mutant fish compared with controls in both the lateral axis (9.2 ± 4.8 Hz) and fore–aft axis (8.7 ± 8.3 Hz). Among recordings from mutants (*n* = 4 cells), two neurons met our criteria as directionally responsive for lateral translation, and one neuron was directionally responsive for fore–aft translation, but modulation depth was low in both directions (Fig. 1*H*, black circles). These data suggest that the bulk of directionally sensitive inputs to vestibulospinal neurons originates from the utricle.

To control for possible compensatory mechanisms in *otogelin* mutants, we also measured vestibulospinal neuron responses to translation after acute chemoablation of inner ear hair cells (Fig. 1*F*, acute hair cell lesion). Similar to the *otogelin* mutants, after acute chemoablation, modulation depth was reduced dramatically in both the lateral axis (16.8 ± 6.1 Hz) and the fore–aft axis (8.4 ± 5.6 Hz; Fig. 1*H*). Across both acute and chronic (*otogelin*) utricle manipulations, modulation depth was strongly affected by lesion condition, but not stimulus direction (two-way ANOVA; main effect of lesion condition: *F*_(2,43)_ = 8.5, *p* = 0.0008; main effect of stimulus direction: *F*_(1,43)_ = 2.1, *p* = 0.16; interaction effect of lesion condition and stimulus direction: *F*_(2,43)_ = 1.6, *p* = 0.22) with lower modulation depth in chronic (Tukey’s *post hoc* test, *p* = 0.004) and acute (Tukey’s *post hoc* test, *p* = 0.014) conditions compared with controls. Acute lesions did not decrease the fraction of neurons directionally responsive to lateral and fore–aft stimuli (100% lateral directional, 75% fore–aft directional; *n* = 4 cells), but the strength of tuning among responsive cells was low (Fig. 1*H*, directional neurons circled). Collectively, our loss-of-function experiments support the conclusion that utricular input is required for normal phasic responses to translation in vestibulospinal neurons.

Together, our data support earlier findings (Liu et al., 2020) that larval zebrafish vestibulospinal neuron activity reflects sensed destabilization originating with the utricle.

#### Larval zebrafish vestibulospinal neurons receive dense spontaneous excitatory and inhibitory synaptic input

**Figure 2.**
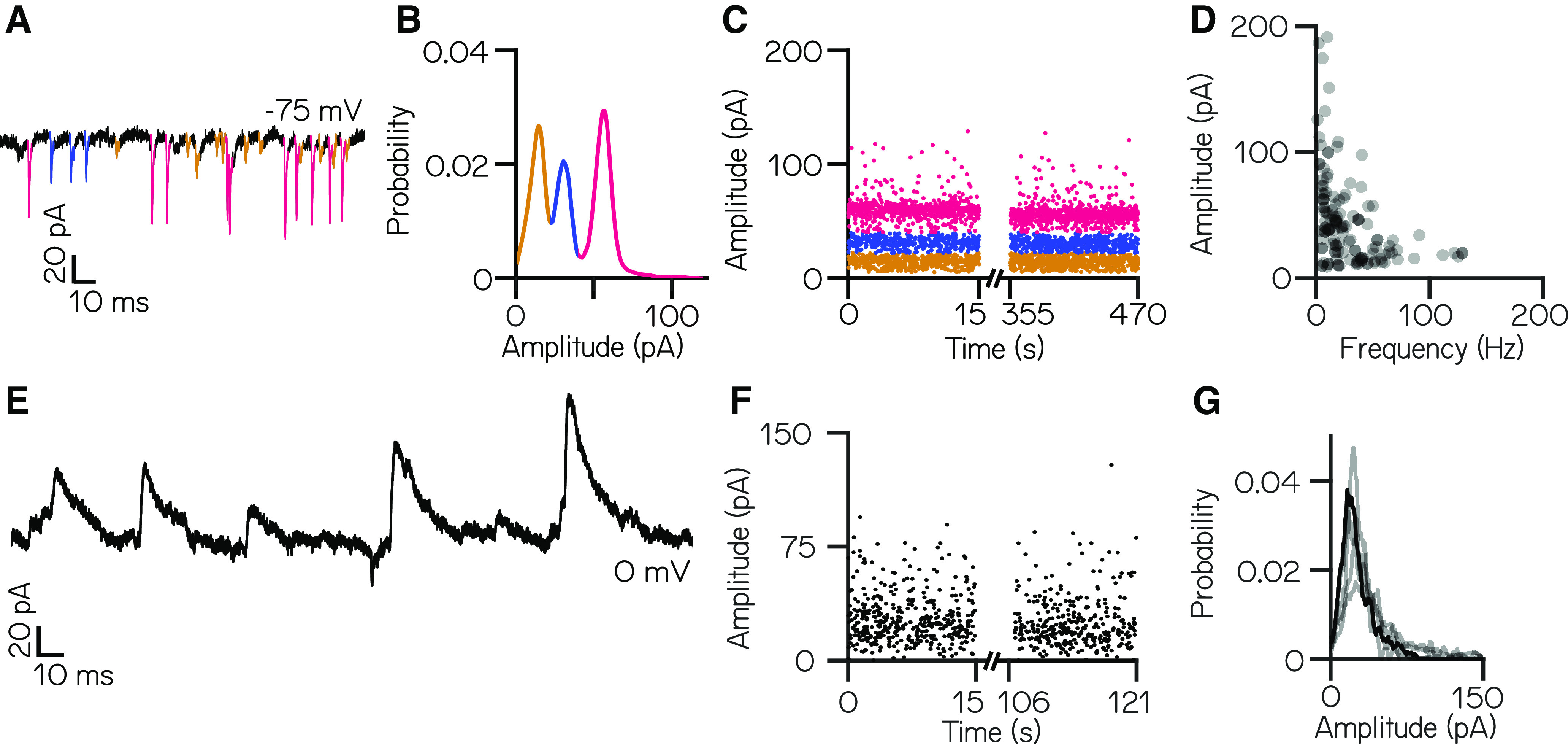
Larval zebrafish vestibulospinal neurons receive dense spontaneous synaptic input. ***A***, Distinct amplitudes (color) in spontaneous EPSCs from a neuron held at –75 mV. ***B***, EPSC amplitudes from a single vestibulospinal neuron show three distinct probability peaks, or “bins” (color). ***C***, EPSC bins are stationary in time. ***D***, EPSC amplitude as a function of frequency for all bins in all vestibulospinal neurons (115 bins, from 35 cells). ***E***, Representative current trace from a vestibulospinal neuron held at 0 mV. ***F***, IPSC amplitudes over time. ***G***, IPSC amplitudes for the example neuron in panels ***E–F*** (black line) and other neurons (gray lines) do not show multiple peaks (*n* = 5).

We next sought to characterize the complement of excitatory synaptic inputs to vestibulospinal neurons at rest. Neurons displayed dense synaptic EPSCs (Fig. 2*A*) with a median frequency of 89.4 ± 41.7 Hz (*n* = 35 neurons). EPSCs showed a wide range of amplitudes (median, 136.0 pA). Amplitude distributions were multimodal in all cells, with distinct peaks visible in a probability distribution (Fig. 2*B*). To characterize these peaks, we assigned EPSCs to amplitude ranges that encompassed each peak in the distribution (Fig. 2*B*, line colors). These bins remained stable over time (Fig. 2*C*). Across our data, neurons had a mean of 3.3 ± 1.0 distinct bins (range, 2-5 bins; 115 bins from 35 cells), with a median event amplitude per bin of 39.5 ± 27.0 pA and a median event frequency per bin of 19.2 ± 20.3 Hz. Bin amplitude and frequency were inversely related (Fig. 2*D*).

We performed a separate set of voltage-clamp experiments to isolate IPSCs. Neurons exhibited spontaneous IPSCs (Fig. 2*E*) with a mean frequency of 22.4 ± 7.4 Hz (range, 13.8–32.5 Hz) and a mean amplitude of 29.1 ± 7.3 pA (range, 22.5–39.2 pA; *n* = 5 neurons). Unlike excitatory inputs, spontaneous inhibitory currents did not have distinct event amplitude peaks (Fig. 2*F*,*G*). We conclude that vestibulospinal neurons receive dense excitatory and inhibitory input at rest.

### EPSC events within the same amplitude bin reflect multiple neuronal inputs

**Figure 3.**
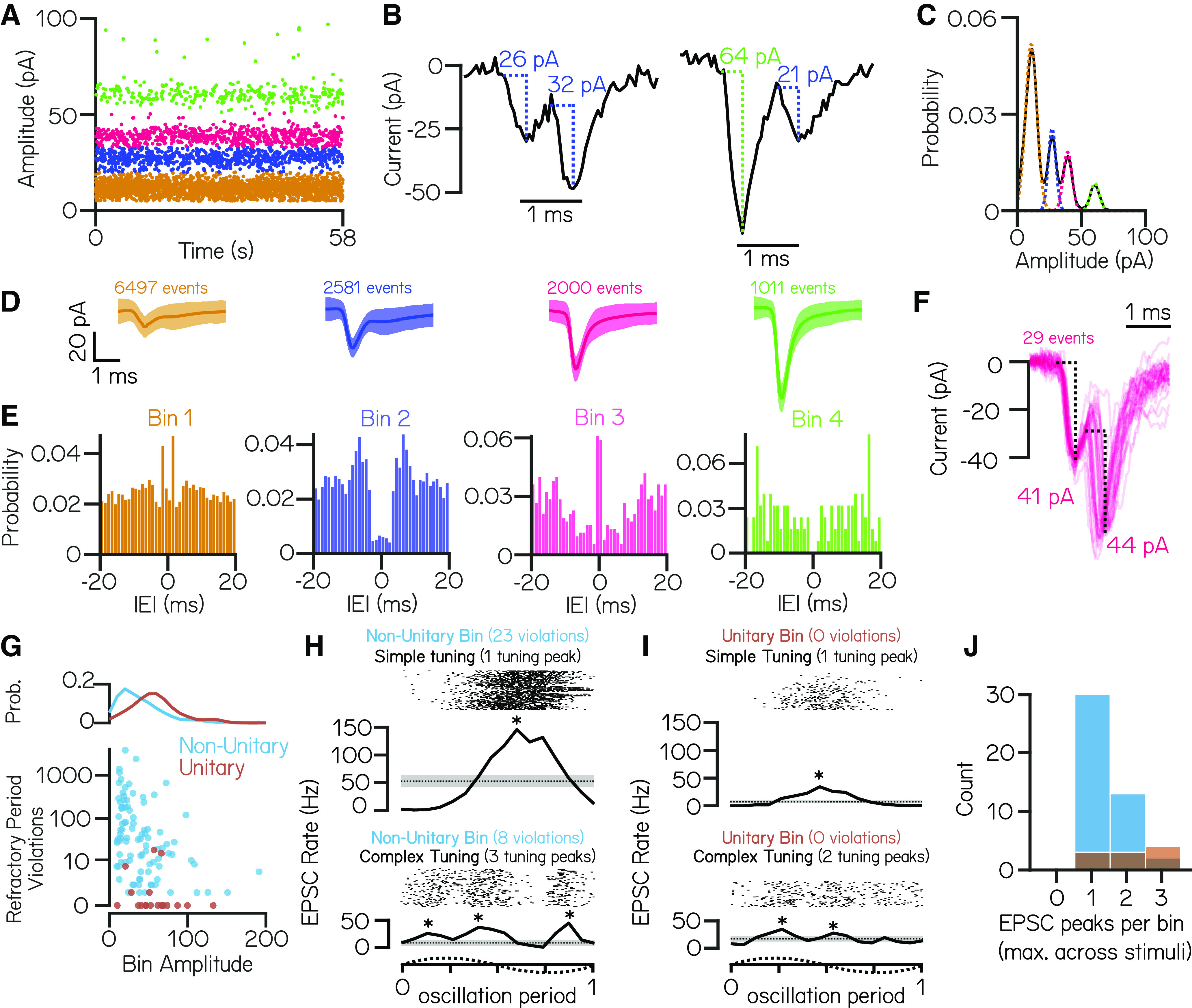
EPSC events within the same amplitude bin predominantly reflect multiple neuronal inputs. ***A***, An example cell with four stable and discrete amplitude bins (colored by bin). ***B***, Example EPSC traces demonstrate events that co-occur within 1 ms. Event pairs are either within-bin (left) or across-bin (right). ***C***, To estimate an upper limit on the expected refractory period violations because of bin overlap, EPSC amplitude distributions were modeled as a sum of individual Gaussians (dashed colored lines). ***D***, Average waveforms from each EPSC bin (±SD; *n* = 6497, 2581, 2000, and 1011 events/bin). ***E***, Autocorrelograms show structure of interevent intervals within an EPSC bin; note peaks near zero in bins 1 and 3, a nonzero valley for bin 2, and a true valley for the high-amplitude bin 4. ***F***, Waveforms from EPSC pairs within bin 3 with latencies <1 ms (*n* = 29 event pairs). The large jitter between peaks is inconsistent with the expected profile of an electrochemical synapse. ***G***, Observed within-bin refractory period violations as a function of bin amplitude for bins assigned as having nonunitary (blue) or unitary (brown) origin. The probability distribution of bin amplitudes is shown above. ***H***, ***I***, EPSC event timing from two example nonunitary (***H***) or unitary (***I***) bins aligned to one oscillation of lateral translation. EPSCs within a single bin can exhibit simple tuning with a single peak in EPSC rate (top) or can have complex tuning with multiple peaks in EPSC rate during oscillation (bottom). Asterisks indicate EPSC tuning peaks. ***J***, Histogram of the number of EPSC rate peaks per amplitude bin during translation (maximum per bin across lateral and fore–aft stimuli) for nonunitary and unitary EPSC bins.

Distinct EPSC bins might reflect input from single VIIIth nerve afferents with different stable resting amplitudes (Bagnall et al., 2008; McElvain et al., 2015). A previous report reached this conclusion based on comparable recordings performed in the light (Liu et al., 2020). To test whether EPSCs within a distinct amplitude bin in our recordings (Fig. 3*A*) derived from a single afferent neuron (a “unitary” origin), we applied refractory period criteria to identify EPSC events that occurred within 1 ms of each other (Fig. 3*B*). We reasoned that if EPSC amplitude bins reflect single afferent inputs, there ought to be no such examples of refractory period violations from within-bin EPSCs (Fig. 3*B*, left).

To account for sources of error (e.g., noisy bin assignations) that might lead us to overestimate within-bin refractory period violations in our data, we compared empirical within-bin refractory period violations to a model estimate of expected violations. Briefly, each EPSC amplitude bin was modeled as a Gaussian distribution centered around the amplitude of each bin, and an upper limit on expected within-bin refractory period violations was set by the overlap in these modeled Gaussians and the frequency of event in each bin (Materials and Methods; Fig. 3*C*). We accounted for the possibility that some trial recordings without refractory period violations were too brief to detect violations in low-frequency EPSC bins and excluded any such bins from further analysis (4 of 115 bins).

We first examined EPSC refractory period violations in an example cell with four amplitude bins (Fig. 3*A*,*C*). Refractory period violations were a minority of events within each bin, evident in the stereotyped shape of the average waveform of EPSCs (Fig. 3*D*). Only one amplitude bin (Fig. 3*D*, Bin 4, green) had no refractory period violations, as expected if bins had a unitary origin. Autocorrelograms of EPSC interevent intervals within an amplitude bin showed that this potentially unitary bin had a distinct valley near 0 ms (Fig. 3*E*), consistent with previous work (Liu et al., 2020). The three remaining bins had more within-bin refractory period violations than estimated by our model of bin overlap, consistent with a nonunitary origin.

We noticed that two bins (Fig. 3*E*, Bins 1 and 3) exhibited significantly more within-bin refractory period violations than expected based on event frequency alone, reflected by a peak near 0 ms in their autocorrelograms; these high-violation bins were not uncommon across bins from all cells (24 of 111 bins). These bins might reflect the presence of compound events (i.e., mixed electrical and chemical synapses) deriving from the same afferent input. Evidence for mixed synapses between the VIIIth nerve and central vestibular nuclei comes from electrophysiology/pharmacology and electron microscopy (Liu et al., 2020) in larval zebrafish, from electrophysiology and electron microscopy in other teleosts (Korn et al., 1977), from electron microscopy in the embryonic chick (Peusner, 1984; Peusner and Giaume, 1994), and from immunofluorescence and electron microscopy in the rat (Nagy et al., 2013). However, waveforms of high-probability compound events did not have the expected shape of a classical electrochemical synapse with an electrical event followed by a stereotyped low-jitter chemical event (Fig. 3*F*). Additionally, electrochemical synapses typically consist of a high-amplitude electrical event followed by a smaller amplitude chemical event (Yao et al., 2014; Liu et al., 2020), not two events of comparable size, as observed here. Finally, these event pairs—while more common than expected by chance—composed a very small percentage of the total events within an amplitude bin (29 of 2000 events in Bin 3). As we did not directly test whether events originated from electrical/chemical origins, our data do not speak to whether vestibulospinal neurons generally receive input from mixed synapses. Nevertheless, we conclude that it is unlikely that the presence of mixed synapses caused us to dramatically underestimate the presence of unitary EPSC bins.

We then turned to see whether our findings generalized across amplitude bins over all cells. As in our example cell, the majority (93 of 111) of amplitude bins had more violations than expected if they originated from a single afferent unit (“nonunitary”; Fig. 3*G*). Compared with nonunitary bins, the few bins that passed our refractory test consisted of higher-amplitude (median, 54.9 pA; unitary vs 31.1 pA nonunitary; Fig. 3*G*) and lower-frequency EPSCs (median, 9.7 Hz unitary vs 21.6 Hz nonunitary). Vestibular afferents are commonly classified with respect to the stereotypy of their interspike intervals, falling into one of two classes: regular or irregular (Goldberg et al., 1990). The interevent intervals of the putative unitary bins were consistent with irregular afferent input (median coefficient of variation, 0.93 ± 0.08).

To further test whether EPSC amplitude bins were consistent with a unitary origin, we investigated the sensory tuning of EPSCs within an amplitude bin using oscillatory translations of the fish during whole-cell recordings. EPSCs within an amplitude bin could be tuned either similarly, with a single peak in EPSC rate (simple tuning), or disparately, with multiple peaks in EPSC rates during an oscillation (complex tuning). As vestibular afferents only respond in a single phase direction (Fernández and Goldberg, 1976a, b; Angelaki and Dickman, 2000), we reasoned that EPSC bins that exhibit multiple peaks in EPSC rate during an oscillation must originate from multiple afferents with disparate directional tuning. Conversely, EPSC bins that exhibit simple tuning to the translation stimulus could be derived either from a single afferent or from multiple converging afferents with the same preferred stimulus direction.

EPSCs from amplitude bins that were determined to be nonunitary by refractory period violations had examples of both simple (30 of 45 bins) and complex (15 of 45 bins) tuning to translation (Fig. 3*H*,*J*), which is consistent with the hypothesis that these EPSCs derive from multiple afferent inputs. Surprisingly, among the EPSC bins determined as putatively unitary by refractory period violations, we still identified bins that had simple (3 of 10) and complex (7 of 10) EPSC tuning (Fig. 3*I*,*J*). This result strongly suggests that the majority of EPSC amplitude bins originate from multiple afferent sources with only 5% of bins being consistent with a single afferent source (no refractory period violations and simple EPSC tuning). We conclude that in our recordings nearly all bins are composed of multiple inputs, but a handful of high-amplitude, low-frequency event bins may be consistent with input from single irregularly firing VIIIth nerve afferents.

### High-amplitude excitatory synaptic inputs originate from ipsilateral ear

**Figure 4.**
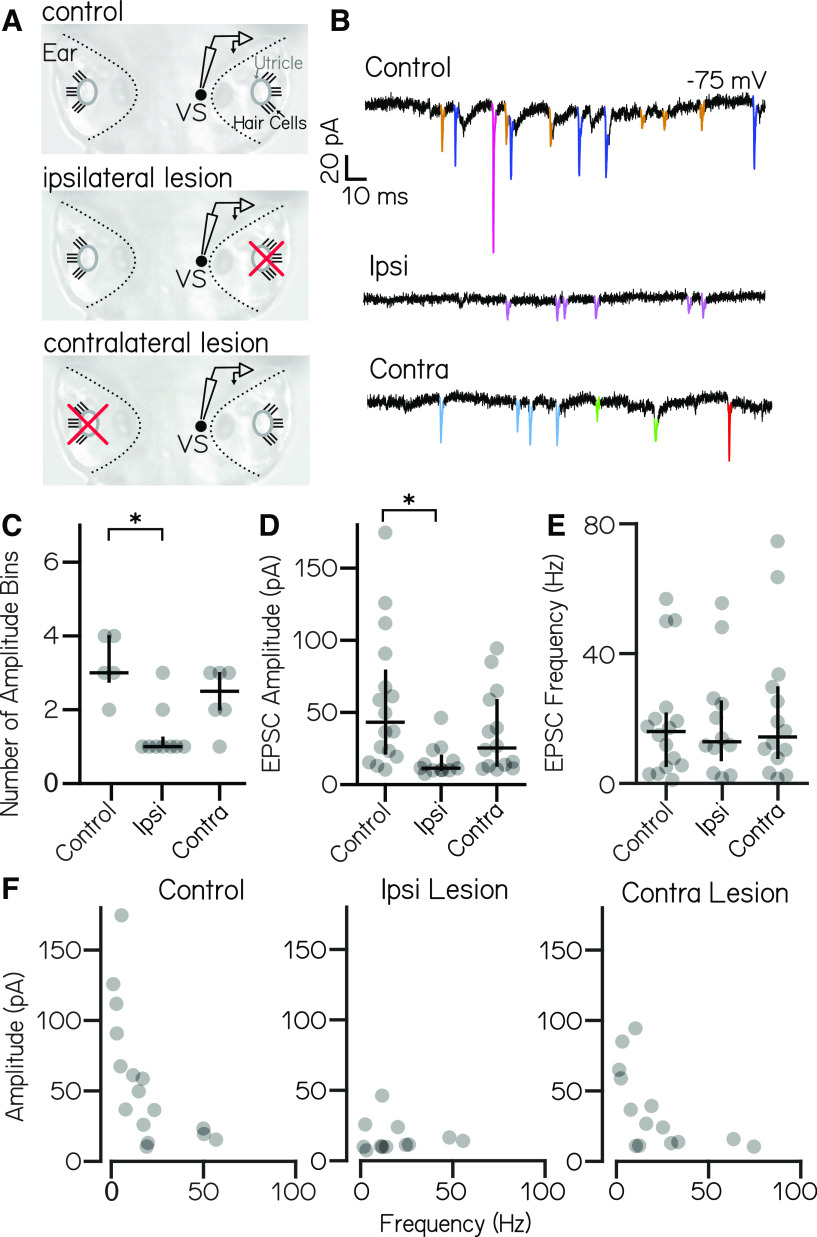
High-amplitude spontaneous excitatory inputs originate in the ipsilateral ear. ***A***, Lesion schematic: the utricle (gray circle) was physically removed (red “x”) either ipsilateral or contralateral to the recorded vestibulospinal neuron (black circle, “VS”). ***B***, Example current traces from neurons held at −75 mV from control (top), ipsilateral (middle), and contralateral (bottom) experiments; EPSCs are in color. ***C***, Number of EPSC amplitude bins per cell [median ± interquartile range (IQR) in black] is decreased after ipsilateral, but not contralateral, lesion. Asterisks indicate statistically significant differences (*p* < 0.05). ***D***, EPSC bin amplitudes (median ± IQR in black) are decreased after ipsilateral, but not contralateral, lesion. ***E***, Frequency of events in EPSC bins (median ± IQR in black) is unchanged after ipsilateral or contralateral lesion compared with control cells. ***F***, EPSC amplitude versus frequency for each bin in control and after ipsilateral/contralateral lesions. High-amplitude bins are lost after ipsilateral lesion.

Our loss-of-function experiments suggest that sensory-driven input to vestibulospinal neurons is predominantly utricular. As each ear contains an utricle, inputs to a given neuron could originate from ipsilateral or contralateral utricular afferents. To differentiate ipsilateral and contralateral contributions, we performed voltage-clamp recordings of spontaneous EPSC activity in vestibulospinal neurons after removing the utricle either ipsilateral or contralateral to the recorded neuron (Figs. 4*A*,*B*). We found that the number of EPSC amplitude bins per cell differed across lesion conditions (Kruskal–Wallis test, *H*(2) = 10.2; *p* = 0.006; Fig. 4*C*). After ipsilateral lesion, neurons had fewer EPSC bins (median, 1 vs 3; *n* = 9 lesions, *n* = 5 controls; Dunn–Sidak *post hoc* test, *p* = 0.006). In contrast, there was no change after contralateral lesion (median, 2.5 bins; *n* = 6; Dunn–Sidak *post hoc* test, *p* = 0.54). Further, the amplitude of EPSC bins also differed across conditions (Kruskal–Wallis test, *H*(2) = 11.7; *p* = 0.003; Fig. 4*D*). EPSC bins after ipsilateral lesion were of lower amplitude than those for controls (median, 11.4 vs 43.2 pA; Dunn–Sidak *post hoc* test, *p* = 0.002), but contralateral lesions did not affect EPSC bin amplitudes (median, 25.4 pA; Dunn–Sidak *post hoc* test, *p* = 0.53). EPSC bin frequency was not changed across lesion conditions (Kruskal–Wallis test, *H*(2) = 0.06, *p* = 0.97; Fig. 4*E*). We conclude that high-amplitude, low-frequency EPSCs derive from ipsilateral inputs (Fig. 4*F*). In contrast, lower-amplitude EPSCs persist after both ipsilateral and contralateral lesions, which might reflect either an extravestibular origin or an incomplete lesion.

### Inhibitory inputs originate with both ipsilateral and contralateral ears

**Figure 5.**
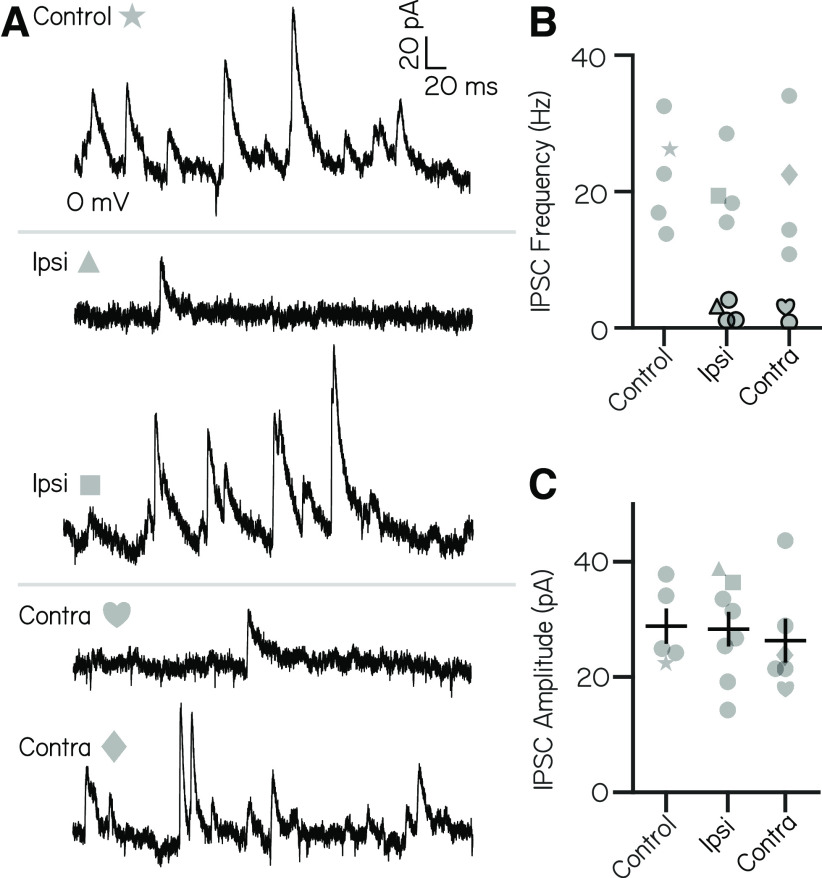
Inhibitory current inputs have ipsilateral and contralateral vestibular sensory origins. ***A***, Control trace from a neuron held at 0 mV shows inhibitory input at rest (top). After ipsilateral (middle) or contralateral (bottom) utricular lesion, some cells experience strong loss of inhibitory currents, while others appear unaffected. ***B***, Distribution of IPSC frequency after ipsilateral or contralateral utricular lesions. Symbols correspond with example neurons in ***A***. Control neurons (*n* = 5) experienced IPSC frequency from 13.7 to 32.5 Hz. After ipsilateral lesions, 4 of 8 neurons experienced IPSC frequencies <2 SDs below control (under 7.5 Hz, symbols outlined in black). After contralateral lesion, 2 of 6 neurons received IPSCs <7.5 Hz. ***C***, IPSC amplitude is unchanged across control and lesion conditions (mean ± SEM).

We then asked whether inhibitory synaptic input onto vestibulospinal neurons originated from the ipsilateral or contralateral ear. We quantified spontaneous IPSCs after ipsilateral or contralateral utricular lesions. In control cells without peripheral lesions, spontaneous IPSCs onto vestibulospinal neurons occurred at a frequency ranging from 13.7 to 32.5 Hz (mean, 22.4 ± 7.4 Hz; *n* = 5 cells). After ipsilateral lesion, we found that IPSC frequency decreased in a subset of neurons, where half of the recorded cells had IPSC frequencies that dropped >2 SDs below the mean frequency of controls (*n* = 4 of 8 cells falling to <7.5 Hz). In contrast, IPSC frequency was comparable to that in control cells in the other half of ipsilateral lesion cells (Figs. 5*A*,*B*). Interestingly, we found that contralateral utricular lesions had a similar effect on the frequency of IPSC input to vestibulospinal neurons. After contralateral utricular lesions, a fraction of cells experienced a drastic reduction in IPSC frequency compared with controls (*n* = 2 of 6 cells falling to <7.5 Hz), while the remaining cells had IPSC frequencies comparable to those of control cells (Fig. 5*A*,*B*). While the reduction in IPSC frequency in a subset of neurons is striking, we note that ipsilateral and contralateral utricular lesion did not have a significant effect on IPSC frequency when looking across all neurons (one-way ANOVA, *F*_(2,16)_ = 1.74; *p* = 0.21), likely because of the sample size and heterogeneous effect of the lesions. Ipsilateral and contralateral lesions did not affect the amplitude of remaining IPSCs (one-way ANOVA, *F*_(2,16)_ = 0.15; *p* = 0.86; Fig. 5*C*). Our data suggest that spontaneous IPSCs onto some vestibulospinal neurons can reflect vestibular input of utricular origin. IPSCs that persist after an ipsilateral or contralateral lesion may derive from the other utricle or nonutricular inhibitory inputs like the cerebellum, or may occur because of an incomplete lesion. Furthermore, our data are consistent with a model where an individual vestibulospinal neuron receives the majority of its inhibition from either the ipsilateral or contralateral ear, rather than a convergence from both ears.

## Discussion

Vestibulospinal neurons are part of a critical and evolutionarily ancient circuit that transforms peripheral sensations of imbalance into postural motor reflexes. Here, we used the larval zebrafish to investigate the source and structure of excitatory and inhibitory synaptic inputs onto zebrafish vestibulospinal neurons. We began by confirming and extending findings from a previous report (Liu et al., 2020). We confirmed that neurons were silent at rest yet capable of finding sustained trains of action potentials on depolarization, and that neurons responded systematically to oscillatory translation in the dark. We replicated the observation that genetic loss of utricular function disrupted these vestibular responses, and extended this finding to acute bilateral lesions of the ear. We validated that vestibulospinal neurons received excitatory synaptic inputs of characteristic amplitude. However, in our recordings, the bulk of these characteristic amplitude bins failed a refractory period test, suggesting a nonunitary origin. We discovered that vestibulospinal neurons also receive strong inhibitory inputs. Finally, we used acute unilateral lesions to show that the loss of ipsilateral input disrupted the highest-amplitude excitatory inputs, and that both ipsilateral and contralateral lesions could disrupt IPSCs. Together, our work both validates a recent characterization of vestibulospinal neurons and extends that work to map circuit-level inputs to larval zebrafish vestibulospinal neurons.

Linear encoding at central vestibular synapses is thought to be important for encoding of head/body position. One way to achieve linear encoding by the maintenance of stable, frequency-invariant EPSC amplitudes (Bagnall et al., 2008). Stable EPSC amplitudes can be instantiated by a number of presynaptic and postsynaptic molecular mechanisms that keep the overall charge transfer across the synapse the same over time (McElvain et al., 2015). A single afferent should therefore have stable excitatory drive over time. If the stable amplitudes of each afferent are different from each other, then inputs onto a postsynaptic neuron should be separable by EPSC amplitude, as was previously reported (Liu et al., 2020). We observe that EPSCs onto vestibulospinal neurons fall into discrete amplitude bins that are stable across time/trials.

However, our data are largely inconsistent with the model that EPSCs within a bin reflect a singular afferent input. Instead, we suggest that a bin consists of input from several afferents with approximately the same stable EPSC amplitude. Each individual afferent maintains stable charge transfer over time, as proposed (Bagnall et al., 2008; McElvain et al., 2015). In this model, the ability to differentiate single afferent inputs while recording postsynaptically is limited by (1) the intrinsic noise of our recordings and (2) the number of inputs converging onto the postsynaptic cell. As our intrinsic noise was low, our preparation likely resulted in more spontaneous and sensory-evoked inputs compared with previous preparations. Importantly, our data nevertheless support a model where afferent synapses onto vestibulospinal neurons achieve linear encoding of head and body movement through stable excitatory drive.

The only major difference between the experimental preparations here and in the study by Liu et al. (2020) was that our recordings were performed exclusively in the dark while theirs were in ambient light. Notably, the previous report focused on analysis of a subset (50%) of recorded vestibulospinal neurons that had one or more amplitude bins whose activity was consistent with a singular origin (M. Bagnall, personal communication). We hypothesize that differences might reflect visual or state-dependent modulation of presynaptic inputs to vestibulospinal neurons. Both visual input (Goldberg et al., 2012) and behavioral state (McArthur and Dickman, 2011) can profoundly impact vestibular neuron activity. Presynaptic inputs to vestibular neurons have previously been shown to reduce neurotransmitter release during sensory gating (Tabor et al., 2018) and are thought to be the site for visually guided motor learning (Lisberger, 1988). In larval zebrafish, such modulation could originate from visually responsive (Mu et al., 2012) dopaminergic neurons that drive vestibulospinal neuron activity when activated (Barrios et al., 2020). We therefore build on and expand models of vestibulospinal circuit organization to offer a tractable way to understand—at a synaptic level—how extravestibular information influences sensed imbalance.

**Figure 6.**
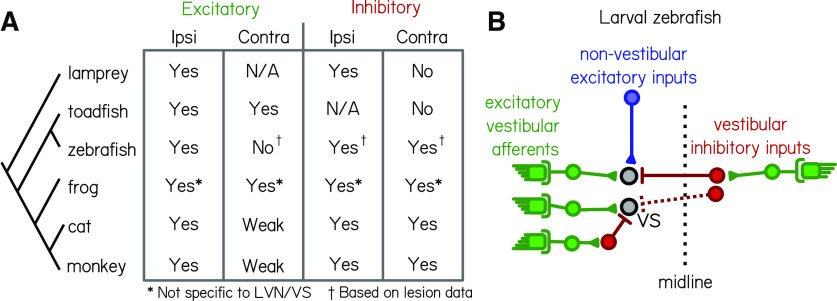
Comparative synaptic architecture of zebrafish vestibulospinal neurons. ***A***, Summary of previous circuit mapping of functional synaptic connections between vestibular afferents and secondary vestibular neurons across species [lamprey (Rovainen, 1979); toadfish (Korn et al., 1977); zebrafish (Liu et al., 2020; current study); frog (Ozawa et al., 1974; Holler and Straka, 2001; Pfanzelt et al., 2008; Malinvaud et al., 2010); cat (Shimazu and Smith, 1971; Uchino et al., 1999, 2001; Kushiro et al., 2000; Ogawa et al., 2000); and monkey (Goldberg et al., 1987; Highstein et al., 1987)]. All characterizations were from vestibulospinal neuron homologs, except for the frog (asterisk) where data were not specific to vestibulospinal neurons in the lateral vestibular nucleus. Connections were determined by afferent activation, except where only afferent lesion data from the current study were available (dagger). ***B***, Vestibulospinal neurons (“VS”, black circles) receive convergent high-amplitude excitatory inputs (green) from irregular afferents originating with the ipsilateral utricle (see also Liu et al., 2020), low-amplitude excitatory inputs (blue) from extravestibular sources and inhibitory inputs (red) from either the ipsilateral or contralateral utricle.

Electrophysiological studies have established a basic map of excitatory and inhibitory vestibular synaptic inputs in a number of vertebrate species (Fig. 6). Vestibulospinal neurons in all species are defined, in part, by receiving excitatory input from the ipsilateral vestibular nerve. There is an existing divide, however, among vertebrate species regarding the role of contralateral vestibular input. Studies in mammals and frogs have shown evidence of contralateral inhibition (Goldberg et al., 1987; Highstein et al., 1987; Uchino et al., 1999, 1986; Holler and Straka, 2001; Malinvaud et al., 2010), which has not been reported in studies of other teleost fish or nonjawed vertebrates (Korn et al., 1977; Rovainen, 1979); conversely, vestibulospinal neurons in many nonmammalian vertebrates receive contralateral excitation (Ozawa et al., 1974; Korn et al., 1977; Holler and Straka, 2001), which is not as commonly seen in mammalian cells (Goldberg et al., 1987; Highstein et al., 1987; Uchino et al., 1999, 2001; Fig. 6*A*). In the cat, where the circuit has been the most carefully mapped, vestibulospinal neurons receive excitatory utricular inputs predominantly from the ipsilateral ear, cross-striolar inhibition from the ipsilateral ear, and commissural inhibition from the contralateral ear (Shimazu and Smith, 1971; Uchino et al., 1999, 2001; Kushiro et al., 2000; Ogawa et al., 2000). The source of inhibitory inputs has been of particular interest when discussing circuit function in mammals, as commissural and cross-striolar inhibition are thought to increase the sensitivity of central vestibular neurons to sensory stimuli (Shimazu and Precht, 1966; Uchino et al., 1997) and to play a role in vestibular compensation (Graham and Dutia, 2001; Bergquist et al., 2008). Further, identifying the neurotransmitters responsible for commissural inhibition in the vestibular nuclei has been an ongoing question (Walberg et al., 1990; Holstein et al., 1999; Bagnall et al., 2007; Pfanzelt et al., 2008; Malinvaud et al., 2010; Popratiloff and Peusner, 2011). Identifying a potential GABAergic or glycinergic source for ipsilateral or contralateral inhibitory inputs onto zebrafish vestibulospinal neurons would facilitate comparing the mechanisms of inhibition both across species and across the different vestibular nuclei.

Our data suggest that individual zebrafish vestibulospinal neurons receive the following: (1) high-amplitude excitatory inputs exclusively from the ipsilateral utricle; (2) utricle-independent low-amplitude excitatory inputs; and (3) inhibitory inputs primarily from either the ipsilateral or contralateral utricle, but likely not from both (Fig. 6*B*). We do not see a change to spontaneous excitatory inputs after contralateral lesions. However, this may reflect the limits of our loss-of-function approach; future experiments could use afferent stimulation to definitively address whether excitatory inputs originate from the contralateral VIIIth nerve. We therefore conclude that zebrafish larvae are closer in their circuit organization to mammals than to other nonmammalian vertebrates, based on the presence of contralateral inhibition and the lack of appreciable contralateral excitation. Circuit mapping as we present here is necessary not only for understanding the logic of this sensory circuit, but for comparing how findings in the zebrafish extend to other species.

We note that our findings are from early larval development; balance control undergoes changes during development (Ehrlich and Schoppik, 2017), which may correspond to circuit-level changes to vestibulospinal inputs in the juvenile and adult. More broadly, zebrafish vestibulospinal neurons can also be compared with those in other species based on their electrophysiological properties. In other species, central vestibular nuclei have been described as containing distinct subtypes of neurons with disparate intrinsic electrophysiological properties (Straka et al., 2005). Zebrafish vestibulospinal neurons, in contrast, appear to be a relatively homogeneous population with properties similar to the silent at rest, tonic-firing subtype of vestibular neurons previously identified in frogs (Straka and Dieringer, 1996; Straka et al., 2004).

As the larval zebrafish has increasingly been used as a useful model for studying vestibular circuit function (Liu et al., 2020; Tanimoto et al., 2022; Beiza-Canelo et al., 2023; Sugioka et al., 2023), development (Ehrlich and Schoppik, 2017, 2019; Liu et al., 2022), and behavior (Zhu et al., 2023), it is necessary that we establish a replicable consensus for the synaptic connections within vestibular circuits in the fish. Here, we validated and extend our understanding of the nature and origin of synaptic inputs onto central vestibulospinal neurons in the larval zebrafish. Though critical for survival, posture control and its underlying vestibulospinal substrates have been less well studied across vertebrate species compared with other vestibular reflex circuits. The larval zebrafish has a vestibulospinal blueprint comparable to that in mammals, despite fundamental differences in locomotor strategies, body plans, and environmental challenges to balance. By studying vestibulospinal neurons and their synaptic inputs in the larval zebrafish, future work may allow us to determine how such an evolutionarily ancient circuit can be modified across species to produce complex strategies for maintaining posture underwater, on land, or in flight. Our work is therefore a major a step toward understanding how sensed imbalance is transformed by these conserved neurons into commands to stabilize posture.

## Synthesis

Reviewing Editor: Nicholas J. Priebe, University of Texas at Austin Decisions are customarily a result of the Reviewing Editor and the peer reviewers coming together and discussing their recommendations until a consensus is reached. When revisions are invited, a fact-based synthesis statement explaining their decision and outlining what is needed to prepare a revision will be listed below. The following reviewer(s) agreed to reveal their identity: Francois Lambert.

SYNTHESIS

Two reviewers evaluated the manuscript “The nature and origin of synaptic inputs to vestibulospinal neurons in the larval zebrafish, Danio rerio” and both reviewers agree that the study provides valuable information for sensory neuroscientists and the vestibular field. They recognized the value of the study both to demonstrate the replicability of experimental results in larval zebrafish across different laboratories and to describe the developmental changes in both excitatory and inhibitory synapses onto central vestibular neurons. Both agreed that the experimental results of are of high quality, presented in clear figures and that the paper is well written.

There were, however, a few suggestions to strengthen the presentation of the work.

1. line 31. “comparison of larval zebrafish neurons across species” This is confusing - do you mean across fish species?

2. line 142. Was the air table pushed manually? Were the oscillations persistent because the table has a resonance frequency? A bit more information about the stimulus delivery consistency would be helpful.

3. line 154. Why settle for arbitrariness of a user-defined spike threshold vs a threshold crossing measure? (eg for dv/dt values)

4. line 160. Please comment on how selection biases were minimized in hand-selected PSC analyses.

5. line 233. For those not familiar with larval zebrafish inner ear - how big is the otolith, and what size forceps were used to remove it?

6. line 256. 21 cells recorded in 4-12dpf larvae. How many larvae to obtain 21 cells? In all the manuscripts please define clearly for all set of experiments the number of cell (n) obtained from a number of animals (N). How important is the development of the vestibular systems, both at a periphery and at a central level between 4 and 12 dpf ? How much variability in results this could introduced? Maybe there is no significant maturation between 4 and 12 dpf but this should be discussed either in this section or in the discussion.

7. line 261-262. Do all neurons show the same discharge pattern? Are they all tonic or some of them exhibited a more transitory discharge dynamic in response to pulse injection? How is the regularity of the discharge frequency? What is the mean discharge frequency? See also the 1rst comment done about the discussion.

8. Fig. 2. Please organize results about IPSCs the same way than EPSCs to allow and a fast comparison between the 2 types of synaptic inputs. Why is the graph Amplitude vs Frequency (Fig. 2D) not provided for IPSCs?

9. line 265. What does it mean to have a mature action potential? (Presumably spike width, which looks strikingly narrow in the figures, but has this been looked at developmentally in zebrafish?)

10. line 302. Add “during body tilts” after “respond phasically”

11. line 328. Replace ‘received’ with something like displayed or had. Neurons produce their own synaptic currents.

12. lines 373-377. Peusner and Giaume 1994 may be relevant to cite here.

13. line 441-445. This sequence is not easy to understand and written in a counter intuitive way compared to Fig. 5B. Writing that the frequency ranging is “increased” in Ipsi condition compared to control is confusing whereas the max frequency in ipsi condition is lower than control (28.5Hz vs 32.5Hz). It looks like there are 3 clusters in the ipsi condition; 4 neurons below 10Hz, 3 neurons around 15-20Hz comparable to the lowest frequency observed in control and 1 neuron at 28Hz. Please simplify the sentence and describe the ipsi results in a more simple way and maybe the addition of a color code or circles surrounding each subpopulation could be used in Fig5B to help the reader to identify subpopulations described in the text.

14. line 445-450. Same comment for IPSC

Fig.5B. like the average of IPSC freq is not shown on the graph I guess there is no significant difference between the 3 conditions. Is it possible to run a statistical test that compare the dispersion of the values, or maybe show some whisker box? Same comment for Fig. 5C.

15. line 452-453. It seems to be true for some neurons (luckily those marked with a symbol for example, ;-)) but the same conclusion than in line 435-436 could be done for neurons showing comparable frequency in ipsi/contra condition to control. Please addressed this point.

16. Discussion, related to results section line 254-268. It is not mentioned either in this result section nor in the discussion if VS neurons exhibited different discharge dynamics in response to current injection (like shown in fig . 1B) and different intrinsic membrane properties like presence of simple or double AHP (like shown in Fig. 1D), comparable to type A/B or phasic/tonic central vestibular neurons described in mammals and in amphibian, respectively. Intracellular recordings done in this study should at least tell if VS neurons exhibit one or several discharge patterns. Even if it’s not the main focus of the paper, it would be interesting to add a few sentences to discuss this point taking account that discharge dynamics and membrane properties are 2 parameters of central vestibular neurons that strongly participate to the tuning of both excitatory and inhibitory vestibulo-motor networks.

16. Line 531-544 and Fig. 6. Fig. 6 will be useful for a lot of people working in the vestibular field and the main conclusions proposed by the authors are appropriate.

The authors appear to be moving toward making a general statement for how their model relates to the vestibular control of posture in general, which may require elaboration. The stabilization of the posture is much more complex than the gaze stabilization and provides more variable motor outputs even in term of vestibulospinal reflexives responses. It directly depends on both the environment (aquatic=reduced gravity sensing and ground perception vs terrestrial=high gravity sensing and ground perception; or aerial environment) and the body organization (axial vs trunk/appendicular; presence or absence of a neck; etc). In addition findings of the present study were established in early larval fish. How much these findings will change during the development, especially with the later appearance of semicircular canals inputs. Finally, concerning the inhibitory part, the authors do not discuss at all about the potential nature (GABA vs Glycine) of described inhibitory inputs on VS neurons (at least for contralateral inputs). All these points should be quickly addressed in the conclusion with a few additional sentences.

17. Line 532. “lower vertebrates”. It is perhaps not useful to employ this wording (lower vs higher vertebrates) anymore in an evolutionary perspectives, especially when we’re talking about motor behavior like posture, fundamental for surviving and commonly shared by all vertebrates lineages. It is only referring to an old, classical and human being-self centered vision of the evolution. There is no lower and higher posture stabilization mechanism but adapted systems to specific body plan organization and environmental constrains (see above). Vestibulospinal pathways in cat allow the animal to turn ventral-side down during the fall of the animal in order to allow him to always hit the ground on its four limbs. Such a capacity is useless for a fish. Moreover cats generally do not like water!! ;-). It is not clear if this sentence is really useful in the discussion.

18. The frequent use of the shortcut transitions “next” or “we next” detract a bit from the otherwise clear writing.

## Author Response

1. line 31. “comparison of larval zebrafish neurons across species” This is confusing - do you mean across fish species?

We appreciate the reviewers’ feedback. We agree this sentence was confusing and have rephrased the sentence to clarify

2. line 142. Was the air table pushed manually? Were the oscillations persistent because the table has a resonance frequency? A bit more information about the stimulus delivery consistency would be helpful.

Yes, the air table was pushed manually. The oscillations persist as long as the experimenter pushed/pulled on handles mounted to the bottom of the table. The table resists displacement and acts as a low-pass filter on the manual oscillations. We have clarified this in lines 146-147 of the text. The stimulus was always delivered by the same experimenter and was quite consistent, as described in line149-154.

3. line 154. Why settle for arbitrariness of a user-defined spike threshold vs a threshold crossing measure? (eg for dv/dt values) For both spike and PSC selection, an initial threshold crossing measure was used to automatically select events. The initial threshold for each trace was automatically set at 15% of the current or voltage range for each trial. On some trials, the threshold needed to be changed by the user if the automatic threshold was obviously inappropriate due to variations in the baseline. All event selections were then manually checked by the experimenter. This allows us to have the highest confidence that we are not missing events or selecting false events. We have clarified these points in the methods in line 157-167.

4. line 160. Please comment on how selection biases were minimized in hand-selected PSC analyses. EPSC and IPSC assignations were made by a first experimenter and then separately checked separately by a second experimenter to ensure that experiments agreed on the selections and that the events had a stereotyped event shape with a fast initial rise and a slow decay. We have added this point to the methods in line 163.

5. line 233. For those not familiar with larval zebrafish inner ear - how big is the otolith, and what size forceps were used to remove it? The otolith at this age is approximately 50 microns in diameter and was removed with size 5CO forceps; these details have been added to the methods at lines 244-246.

6. line 256. 21 cells recorded in 4-12 dpf larvae. How many larvae to obtain 21 cells? In all the manuscripts please define clearly for all set of experiments the number of cell (n) obtained from a number of animals (N).

For all experiments, only a single neuron was recorded from each fish. For the entirety of the manuscript (n) is equal to (N). We have made this clear in Line 268.

How important is the development of the vestibular systems, both at a periphery and at a central level between 4 and 12 dpf ? How much variability in results this could introduced? Maybe there is no significant maturation between 4 and 12 dpf but this should be discussed either in this section or in the discussion.

While we did include data from fish from a wider range of ages, the majority of fish from which cells were recorded were closer in age, between 4 and 7 dpf. We have clarified this in the methods (Lines 115-117). We have analyzed the results with age as a variable and did not see a significant effect of age on any results, therefore we feel confident in combining the data across this developmental range (we’ve added this point in Results, Line 268).

7. line 261-262. Do all neurons show the same discharge pattern? Are they all tonic or some of them exhibited a more transitory discharge dynamic in response to pulse injection? How is the regularity of the discharge frequency? What is the mean discharge frequency? See also the 1st comment done about the discussion.

Qualitatively, we observed that all cells had tonic firing - we never saw cells display a transitory, phasic spiking response to current injection if the current step was sufficiently above the cell’s rheobase. We have added this observation to the results (Line 278). As these experiments were designed only to test the cell’s rheobase, we do not have sufficient data to quantify the discharge regularity or frequency in a manner that would be comparable across all recorded cells.

8. Fig. 2. Please organize results about IPSCs the same way than EPSCs to allow and a fast comparison between the 2 types of synaptic inputs. Why is the graph Amplitude vs Frequency (Fig. 2D) not provided for IPSCs?

We understand the reviewers’ desire to have the EPSC and IPSC analyses align as to make easy comparisons between the two. We have chosen not to make this change because it would require us to add an extra panel to the IPSC analysis to make the panels even, and we believe that adding the comparable panel (Frequency vs Amplitude) would be 1) not informative and 2) possibly confusing to readers for the following reasons:

The graph of Frequency vs Amplitude is not provided for IPSCs because the analysis in Fig. 2D for EPSCs is for each amplitude bin (not per cell). As the IPSCs do not display amplitude bins, it is impossible to present an equivalent analysis. The closed we could come would be to do IPSC analysis cell-by-cell which would result in readers interpreting each dot in Fig. 2D as a single cell.

Further, if we were to plot IPSC frequency vs amplitude per cell, there is very little variation in the mean frequency and amplitude between the 5 recorded cells (see Line 356-357), so there is no meaningful relationship between frequency and amplitude to be shown on this graph. Given the incomparability and to maximize clarity we have decided not to plot the data as suggested.

9. line 265. What does it mean to have a mature action potential? (Presumably spike width, which looks strikingly narrow in the figures, but has this been looked at developmentally in zebrafish?)

We have removed the word “mature” from the sentence as the development of zebrafish action potential waveform has not been sufficiently characterized, and the point is not important for results.

10. line 302. Add “during body tilts” after “respond phasically”

This addition has been made.

11. line 328. Replace ‘received’ with something like displayed or had. Neurons produce their own synaptic currents.

We appreciate the reviewers’ careful reading. We have replaced ‘received’ with other language at several points in the results (lines 346, 356) specifically when the sentence referred to receiving current, but have kept ‘received’ in sentences that referenced the cells receiving synaptic inputs.

12. lines 373-377. Peusner and Giaume 1994 may be relevant to cite here.

We appreciate the reviewers suggestion; this and other citations relevant to mixed synapses in the chick have been added (Line 395).

13. line 441-445. This sequence is not easy to understand and written in a counter intuitive way compared to Fig. 5B. Writing that the frequency ranging is “increased” in Ipsi condition compared to control is confusing whereas the max frequency in ipsi condition is lower than control (28.5Hz vs 32.5Hz). It looks like there are 3 clusters in the ipsi condition; 4 neurons below 10Hz, 3 neurons around 15-20Hz comparable to the lowest frequency observed in control and 1 neuron at 28Hz. Please simplify the sentence and describe the ipsi results in a more simple way and maybe the addition of a color code or circles surrounding each subpopulation could be used in Fig5B to help the reader to identify subpopulations described in the text.

We thank the reviewers for their feedback. We have changed the wording in this section (Lines 462-473) to be clearer by removing references to the frequency range increasing. In the figure, we have added black symbol outline to indicate cells that fell below 7.5 Hz (2 S.D. below the mean of the control) and have amended the text to reflect this change.

While we appreciate the desire to identify patterns / groups in the data, we respectfully disagree with the reviewers that there are 3 meaningful clusters to the ipsilateral data - with n=8 cells, we simply do not have enough data to evaluate this hypothesis.

14. line 445-450. Same comment for IPSC Fig.5B. like the average of IPSC freq is not shown on the graph I guess there is no significant difference between the 3 conditions. Is it possible to run a statistical test that compare the dispersion of the values, or maybe show some whisker box? Same comment for Fig. 5C.

The mean of IPSC frequency is not shown on the graph because central tendency is not an accurate measure for describing the data in the lesion conditions (particularly ipsilateral lesion which looks roughly bimodal). The reviewers are correct that when comparing means between the conditions there is no significant difference, and we have added this point in the results (Lines 470-473). While it would be an appropriate test to run, we do not have enough data to test whether the dispersion of the values is statistically significant.

15. line 452-453. It seems to be true for some neurons (luckily those marked with a symbol for example, ;-)) but the same conclusion than in line 435-436 could be done for neurons showing comparable frequency in ipsi/contra condition to control. Please addressed this point. We have addressed this point and acknowledged possible other sources of inhibition in lines 476-478.

16. Discussion, related to results section line 254-268. It is not mentioned either in this result section nor in the discussion if VS neurons exhibited different discharge dynamics in response to current injection (like shown in fig . 1B) and different intrinsic membrane properties like presence of simple or double AHP (like shown in Fig. 1D), comparable to type A/B or phasic/ tonic central vestibular neurons described in mammals and in amphibian, respectively.

Intracellular recordings done in this study should at least tell if VS neurons exhibit one or several discharge patterns. Even if it’s not the main focus of the paper, it would be interesting to add a few sentences to discuss this point taking account that discharge dynamics and membrane properties are 2 parameters of central vestibular neurons that strongly participate to the tuning of both excitatory and inhibitory vestibulo-motor networks.

All vestibulospinal neurons we recorded had similar tonic firing responses, and similar intrinsic membrane properties (though our measures were not intended to be comprehensive).

Qualitatively, these neurons appear to have single AHPs. Therefore we have no evidence to support that the zebrafish vestibulospinal nucleus has two subtypes comparable to the A/B or phasic/tonic subtypes that have been found in other vestibular nuclei. We have added this point to the discussion (Lines 570-576).

17. Line 531-544 and Fig. 6. Fig. 6 will be useful for a lot of people working in the vestibular field and the main conclusions proposed by the authors are appropriate.

The authors appear to be moving toward making a general statement for how their model relates to the vestibular control of posture in general, which may require elaboration.

The stabilization of the posture is much more complex than the gaze stabilization and provides more variable motor outputs even in term of vestibulospinal reflexives responses. It directly depends on both the environment (aquatic=reduced gravity sensing and ground perception vs terrestrial=high gravity sensing and ground perception; or aerial environment) and the body organization (axial vs trunk/appendicular; presence or absence of a neck; etc).

We have added a general statement addressing this point at Lines 583-590.

In addition findings of the present study were established in early larval fish. How much these findings will change during the development, especially with the later appearance of semicircular canals inputs.

We have added mentioned this point in the discussion (Line 568).

Finally, concerning the inhibitory part, the authors do not discuss at all about the potential nature (GABA vs Glycine) of described inhibitory inputs on VS neurons (at least for contralateral inputs).

We do not have any data/prior literature in the fish to suggest whether these inputs are GABA/ glycinergic, but we agree it would be an interesting point to follow up on and have added this to the discussion in Line 550-556.

All these points should be quickly addressed in the conclusion with a few additional sentences.

18. Line 532. “lower vertebrates”. It is perhaps not useful to employ this wording (lower vs higher vertebrates) anymore in an evolutionary perspectives, especially when we’re talking about motor behavior like posture, fundamental for surviving and commonly shared by all vertebrates lineages. It is only referring to an old, classical and human being-self centered vision of the evolution. There is no lower and higher posture stabilization mechanism but adapted systems to specific body plan organization and environmental constrains (see above).

Vestibulospinal pathways in cat allow the animal to turn ventral-side down during the fall of the animal in order to allow him to always hit the ground on its four limbs. Such a capacity is useless for a fish. Moreover cats generally do not like water!! ;-). It is not clear if this sentence is really useful in the discussion.

We appreciate and agree with the reviewers’ opinion that this phrasing was not useful and have changed the wording (Lines 80, 535 & 564).

19. The frequent use of the shortcut transitions “next” or “we next” detract a bit from the otherwise clear writing.

We thank the reviewers for this helpful feedback. We have cut out several instances of these transitions from the text.
